# McConnell in Shock

**DOI:** 10.7759/cureus.15819

**Published:** 2021-06-21

**Authors:** Joana Silva Marques, Rui Correia, Joana Correia, Gonçalo Ferreira, Nuno Monteiro

**Affiliations:** 1 Internal Medicine, Centro Hospitalar Tondela-Viseu, Epe, Viseu, PRT; 2 Cardiology, Centro Hospitalar Tondela-Viseu, Epe, Viseu, PRT

**Keywords:** mcconnell sign, massive pulmonary embolism, obstructive shock, point-of-care-ultrasound, fibrinolysis

## Abstract

The ideal approach to hemodynamically unstable patients requires the quick identification of the type of shock and its etiology. This can be a challenge in critically ill patients due to the limited information, the wide number of differential diagnosis and the need for fast intervention. Point-of-care ultrasound (POCUS) is a non-invasive, low-cost, real-time and reliable tool used to rapidly and accurately assess hemodynamically unstable patients at the bedside. It can support diagnosis, tailor therapy and guide further workup, especially in patients deemed too unstable to undergo other imaging studies. The authors describe the case of a patient in obstructive shock due to pulmonary embolism, in which McConnell sign was identified by bedside echocardiography, before lab tests and pulmonary computerized tomography angiogram results were obtained

## Introduction

Shock is a life-threatening condition characterized by a severe mismatch between the supply and demand of oxygen. Although this can lead to multiorgan failure, different therapeutic measures are needed depending on the type and etiology of shock. 

Recognizing the possible causes of shock in the critically ill patient in the emergency department (ED) is difficult due to limited information and extensive differentials [1]. Point-of-care ultrasound (POCUS) is a low-cost, real-time, noninvasive tool that allows a quick examination. It is useful to evaluate undifferentiated shock, limiting differential diagnosis and tailoring therapy and workup [1].

The authors present the case of an ED patient with obstructive shock identified by POCUS before other exams.

## Case presentation

An 82-year-old male presented to ED with sudden onset of drowsiness and hypotension. Previous medical history was significant for hypertension, dyslipidaemia, obesity, liver steatosis, benign prostatic hyperplasia and severe acute respiratory syndrome coronavirus 2 (SARS-CoV-2) infection two months earlier. Current medication included furosemide, clopidogrel, perindopril, indapamide, metformin, silodosin, and iron.

At admission, the patient was normotensive (blood pressure (BP): 115/70 mmHg), but tachycardic (heart rate (HR): 120 bpm); had a respiratory rate of 22 cpm and showed signs of poor peripheral perfusion. He had otherwise normal cardiac and pulmonary examination, no signs of deep venous thrombosis such as unilateral leg swelling or calf tenderness. Arterial blood gas (ABG) revealed respiratory alkalemia, hypoxemia and hyperlactacidemia (pH 7.50, pCO_2_ 29mmHg, pO_2_ 48mmHg, SatO_2_ 87%, lactate 3.8mmol/L). He started fluid-challenge and oxygen therapy, while further workup was ordered. 

On re-evaluation, his BP dropped (70/41 mmHg), maintained tachycardia (110 bpm) and hypoxemia (pO_2 _50 mmHg). Electrocardiography showed sinus tachycardia with 120bpm.

Bedside ultrasound was performed showing right ventricular (RV) overload, severe systolic disfunction and free wall hypokinesis with apical sparing on echocardiography (McConnell sign) (Video 1).

**Video 1:** McConnell sign.

A subsequent computerized tomography pulmonary angiogram (CTPA) was obtained confirming bilateral massive pulmonary emboli and infarction of superior lobe of the left lung. Laboratory workup was significant for high troponin 3056.16 ng/L (range 37.50-80.35), N-Terminal Pro-Brain Natriuretic Peptide (NTproBNP) concentration 1778 pg/mL (ref range <125 pg/mL), D-dimer 17436 ng/mL (range 45.0-250.0).

He started fibrinolytic treatment with alteplase 100 mg over two hours and norepinephrine.

He was admitted to the coronary intensive care unit.

Twenty-four hours later his systolic blood pressure (SBP) was 105-120 mmHg, HR 75 bpm and lactates 1 mmol/L. Bedside ultrasound showed no right heart overload, tricuspid annular plane systolic excursion 20 mm, good left ventricle systolic function and no inferior vena cava (IVC) distention with inspiratory collapse.

He was discharged 11 days later with hemodynamic stability, with no dyspnea on exertion or hypoxemia. 

## Discussion

Pulmonary embolism (PE) is a frequently underestimated, underdiagnosed, and undertreated disease [2]. It is a cardiovascular emergency with mortality of 15%-25% if complicated with unstable hemodynamics [2-3]. Quick diagnosis of PE is essential because rapid management can decrease mortality but is frequently confounded by nonspecific clinical presentation [2]. 

Diagnosis and early treatment of PE can be lifesaving. CTPA is the gold standard for PE diagnosis but often unsuitable for unstable patients; An imaging modality that can be rapidly performed at the immobile and critically ill patient's bedside is needed [3]. Bedside critical care ultrasound is a real-time exam with many advantages, as it is rapidly performed and non-invasive [3].

Echocardiographic findings that support the diagnosis of acute PE can be indirect as enlarged RV in parasternal long-axis view, dilated RV with basal RV/left ventricle ratio>1.0, McConnell sign, flattened intraventricular septum in parasternal short-axis view, distended IVC with diminished inspiratory collapsibility in subcostal view; or direct identification of right heart mobile thrombus. Generally, indirect signs have poor sensitivity (29-56%) except McConnell sign [1, 4].

In obstructive shock due to acute PE, echocardiograph images will demonstrate signs of right heart strain or dysfunction and sudden pressure on RV can cause ventricular wall to balloon outward and display the McConnell sign [5]. Thus, McConnell’s sign is characterized by hypokinesis of RV mid-free wall with preserved apical contractility seen in apical four-chamber view. It is seen in only 20% of PE with hemodynamic instability cases. In the original study, it was observed a 77% sensitivity and 94% specificity. Over time, other studies have validated the results and came to a similar conclusion. [4, 6, 7]. 

According to the 2019 Guidelines of European Society of Cardiology in “hemodynamically compromised patient with suspected PE, unequivocal signs of RV pressure overload, especially with more specific echocardiographic findings (60/60 sign, McConnell sign, or right heart thrombi), justify emergency reperfusion treatment for PE if immediate CT angiography is not feasible in a patient with high clinical probability and no other obvious causes for RV pressure overload” [4].

This case demonstrates the successful application of bedside ultrasound in the diagnosis of massive PE in a patient with hemodynamic instability. Although thrombolytics were given immediately after confirmation of PE by CTPA, the McConnell sign also suggested the diagnosis.

## Conclusions

PE was diagnosed using bedside echocardiogram, observing the McConnell sign that is rare but specific and confirmed with CTA of the thorax. Further validation and real-time implementation of this low-cost modality could facilitate the decision to implement thrombolytics for unstable patients with massive pulmonary embolism who cannot undergo formal radiographic evaluation.

## References

[1] Chung-Esaki H, Knight R, Noble J, Wang R, Coralic Z (2012). Detection of acute pulmonary embolism by bedside ultrasound in a patient presenting in PEA arrest: a case report. Case Rep Emerg Med.

[2] Comert SS, Caglayan B, Akturk U (2013). The role of thoracic ultrasonography in the diagnosis of pulmonary embolism. Ann Thorac Med.

[3] Zhu R, Ma XC (2017). Clinical value of ultrasonography in diagnosis of pulmonary embolism in critically ill patients. J Transl Int Med.

[4] European Society of Cardiology. (2019 (2019). European Society of Cardiology. Guidelines on Acute Pulmonary Embolism (Diagnosis and Management of). https://www.escardio.org/Guidelines/Clinical-Practice-Guidelines/Acute-Pulmonary-Embolism-Diagnosis-and-Management-of.

[5] Allen J, Miller BR, Vido MA, Makar GA, Roth KR (2020). Point-of-care ultrasound, anchoring bias, and acute pulmonary embolism: a cautionary tale and report. Radiol Case Rep.

[6] Kansara T, Quesada F, Park H, Ghosh K, Saeed M (2019). McConnell's sign still holds its value: a lesson learned from two cases. Cureus.

[7] McConnell MV, Solomon SD, Rayan ME (1996). Regional right ventricular dysfunction detected by echocardiography in acute pulmonary embolism. Am J Cardiol.

